# Subacute myoclonic measles encephalitis – An opportunistic HIV-associated infection

**DOI:** 10.3389/fncel.2023.1113935

**Published:** 2023-04-04

**Authors:** Luminita Ene, Dan Duiculescu, Roxana Radoi, Mihaela Lazar, Gratiela Tardei, Eugenia Ungureanu, Simona Ruta, Harry V. Vinters, Scott Letendre, Igor Grant, Ronald J. Ellis, Cristian L. Achim

**Affiliations:** ^1^“Dr. Victor Babes” Hospital for Infectious and Tropical Diseases, Bucharest, Romania; ^2^Cantacuzino Institute, Bucharest, Romania; ^3^Carol Davila University of Medicine and Pharmacy, Bucharest, Romania; ^4^Ştefan S. Nicolau Institute of Virology, Bucharest, Romania; ^5^University of California, Los Angeles, Los Angeles, CA, United States; ^6^University of California, San Diego, La Jolla, CA, United States

**Keywords:** HIV, measles encephalitis, myoclonus, opportunistic infection (OI), children and adolescents

## Abstract

**Introduction:**

An unusual cluster of myoclonic epilepsy was observed in a Romanian pediatric HIV cohort concurrent with measles outbreaks. We describe this particular form of subacute measles encephalitis (SME) in a group of HIV-infected children and adolescents with severe immunosuppression.

**Methods:**

This is a single-center study, starting in 1997 and covering 4 measles outbreaks in Romania. The presumptive diagnosis of subacute myoclonic measles encephalitis (SMME) was based on: (1) epidemiological data, previous measles episode or presumed contact with measles virus (MV), (2) clinical presentation with initial localized myoclonic jerks with rapid extension and subsequent motor deficit with preserved mental status, and (3) neuroimaging studies revealing cortical gray matter lesions. Definitive diagnosis was based on a neuropathological exam and immunohistochemistry of brain tissues, and measles RNA detection in the cerebrospinal fluid (CSF).

**Results:**

Thirty-six patients were diagnosed with a particular form of SME during consecutive measles outbreaks in Romania: 1996–1998 (22); 2005–2008 (12); 2010–2011 (1) and 2016-2018 (1). Most children were born in the late 80s and had parenterally acquired HIV infection in early childhood. Before the episode of SMME, 11 patients had confirmed measles, while the rest, without typical rash, had a respiratory tract infection and/or presumed previous measles contact. In all patients, the clinical onset was sudden, with unilateral myoclonus. MRI findings revealed mainly focal cortical gray matter lesions. Neurologic symptoms progressed rapidly to coma and death in most patients. Three patients survived SMME, they had higher CD4 count at onset, slower progression of neurological symptoms, and benefit of immune recovery with cART. Immunocytochemistry studies revealed MV in the brain with a pattern suggesting an ascending viral neural infection. MV was isolated from CSF in 7 out of 8 patients. Sequence analysis of MV RNA from both nasopharyngeal swabs and CSF was available for one patient with similar N-450 strain characteristics.

**Conclusion:**

During an outbreak of measles, neurological manifestations, especially myoclonus in immunosuppressed patients, can be related to measles even in the absence of an acute episode. This particular form of subacute myoclonic measles encephalitis is an opportunistic fatal disease. Immune recovery due to effective antiretroviral treatment might increase survival.

## Introduction

An alarming measles outbreak was ongoing in the European region for the last few years with record cases in ECDC (2018). In this context, immune-suppressed patients are at high risk for measles-related complications. Among neurological complications of measles, subacute measles encephalitis (SME) or measles inclusion body encephalitis (MIBE) was described in immunosuppressed patients regardless of their age. There are few case reports of SME in HIV-infected patients (Mustafa et al., 1993, Vigliano et al., 1995, Budka et al., 1996, Poon et al., 1998) and a case series in South Africa (Albertyn et al., 2011, Hardie et al., 2013) and a more recent report from the last measles epidemic (Rodriguez et al., 2020). SME usually presents with progressive neurological deterioration (altered mental status, lethargy, intractable focal motor seizures or epilepsia partialis continua and weakness) with no effective treatment and high mortality rates.

At the end of the 1980s Romania experienced the world’s largest pediatric epidemic of parenteral nosocomial transmission of HIV (Kozinetz et al., 2000). The majority of patients from the Romanian Pediatric Cohort, now young adults, were infected with HIV-1 after birth in their first year of life, between 1987 and 1990. Within this cohort is a subgroup of severely immunosuppressed patients due to either late presentation or multi-drug resistant HIV, from poor adherence to cART regimens.

Since 1990 in Romania HIV-infected children experienced several measles epidemics in 1996–1998 (Hennessey et al., 1999, Pistol et al., 2003) and 2005–2006 (Ion-Nedelcu et al., 2007, Waku-Kouomou et al., 2007), 2010–2012 (Muscat et al., 2009, Stanescu et al., 2010, Necula et al., 2013), and 2016–2018 (Kriss et al., 2017, Orosz et al., 2018).

Here we describe a particular form of SME that occurred during consecutive measles outbreaks starting 1997–1998 in an HIV reference center, among HIV-infected children and young adults from the Romanian HIV Pediatric Cohort.

## Materials and methods

We describe a group of HIV-1-infected children and young adults that developed subacute encephalitis with the presence of myoclonic jerks as a common pattern during consecutive measles outbreaks. All patients were followed at “Dr. Victor Babes” Hospital for Infectious and Tropical Diseases (VBH), a reference center for HIV in Romania. The study was approved by the Ethics Committee of VBH.

As all patients shared a common pattern, we propose the term subacute measles myoclonic encephalitis (SMME) with the following case definition (1) epidemiological data: previously demonstrated measles episode or presumed contact with measles virus (MV) 2-6 months before the onset of neurological complication; (2) clinical presentation with initially localized myoclonic jerks with rapid extension and subsequent motor deficit and initially preserved mental status; and (3) neuroimaging studies revealing cortical gray matter lesions, hyperintense T2 cortico-subcortical and hypointense T1 lesions; (4) definitive diagnosis was based on neuropathological exam and immunohistochemistry of brain tissues, as well as on the presence of MV in cerebrospinal fluid (CSF).

Clinical and neuroimaging (CT or MRI) data were collected to describe the neurological complication and laboratory data for the evaluation of measles antibody dynamics and assess the moment of HIV infection. EEG studies were available for only a few of the patients as referral to another hospital was difficult due to their severe neurological condition.

Data regarding vaccination and measles diagnosis (if available) were reviewed. In children with undiagnosed measles infections, presumed measles contact was recorded based on parents’ or legal guardians’ information. HIV-infection monitoring was performed only by CD4 count during the 1997–1999 measles epidemic, and both by CD4 and HIV-RNA from plasma and cerebrospinal fluid starting in 2006. HIV RNA detection limit varied over time from 400 to 50 copies/ml. Serological and where possible molecular screening panels included cytomegalovirus (CMV), Toxoplasma gondii, herpes simplex virus (HSV) 1 and 2, Epstein-Barr virus (EBV), JCV, rubella, West-Nile and venereal disease research laboratory (VDRL). MV antibodies were measured using measles antibody assays IgM + IgG (Novum Diagnostica, Dietzenbach, Germany), at the onset of SMME and when available on stored serum within 6–12 months before. CSF routine analyses, and bacterial and fungal cultures were performed for each patient.

MV isolation attempts were done from CSF. Two polymerase chain reaction (PCR) “in house” tests for measles RNA were adapted after a previous publication (Plumet and Gerlier, 2005). The first test was a classic PCR, the second was a real-time PCR using Roche FastStart DNA Master SYBR green I and Light Cycler 2.0 assays. The primers used for both PCRs targeted the N gene. They amplified a 152 bp fragment. The positive control was a suspension of attenuated live virus (10,000 TCID50/ml). Both PCR assays detected a minimum of 100 TCID50/ml from the live attenuated virus and variable concentrations of the wild type of virus from other specimens (i.e., pharyngeal swabs from patients with confirmed measles).

Clinical specimens such as nasopharyngeal swabs and cerebrospinal fluid (CSF), were used for viral RNA extraction directly from 150 μl of sample using the Nucleospin Viral RNA kit (Macherey, Germany) according to the manufacturer’s instructions. Each RNA sample was stored at-70 °C until amplification by real-time reverse transcription PCR (qRT-PCR). Sequencing of the 450 nt of the C-terminal region of the measles virus nucleoprotein gene (N-450) was attempted to determine the genotype by using the one-step RT-PCR kit according to the manufacturer’s protocol (QIAGEN OneStep RT-PCR Kit, Hilden, Germany) (Lazar et al., 2019).

Autopsy brain tissues from all 9 cases with available brain tissues, were processed for histology and neuropathologic examination. Immunohistochemistry was performed on 5-micron sections of paraffin-embedded tissues using a measles monoclonal antibody (Chemicon EMD Millipore cat#MAB8905/Sigma-Aldrich, Clones CV1, CV4).

Statistical analyses were performed using paired samples *t*-test was used to compare HIV RNA log values from plasma and CSF, and an independent *t*-test was used to compare survival in patients stratified by CD4 count.

## Results

A case series of 36 patients were diagnosed with a particular form of SME during consecutive measles outbreaks in Romania: 1996–1998: 22 patients and 2005–2008: 12 patients, 2010–2011: one patient, and 2016–2018 one patient. The general characteristics of study participants are shown in Table 1.

**TABLE 1:** General characteristics of patients diagnosed with SMME during consecutive measles outbreaks.

		Group 1997-1998	Group 2005-2008	2010-2011	2016-2017
No of patients		22	12	1	1
Girls/boys		9/13	4/6	0/1	0/1
Age at SMME diagnosis	Median Limits	9.1 7.3–10.6	16.9 (6.9–20.0)	21	29
HIV-1 transmission route	Parenteral Vertical	22 0	11 1	1 0	1 0
Time since HIV diagnosis	Median years (limits)	2.1 (0–8.7)	6.5 (0.5–11.9)	12	22
	Concomitant diagnosis	4	0	–	–
HIV classification	Clinical B C	12 10	4 8	1 0	0 1
CD4 count at diagnosis CD4%	Median, limits(lf/mmc) Median, limits	112 (15–409) 7 (1.4–17.8)	21 (1–231) 2 (0.2–14)	92 5.8	40 1.5
HIV-1 RNA log10 c/ml Median (limits)	Plasma CSF	N/A N/A	4.2 (2.25–6.0) 2.6 (2.05–2.89)	5.11 3.74	3.95 <1.60
Evidence of measles vaccination	1 dose vaccine	4	4	-	–
	2 doses of vaccine	3	2	-	1
	Not vaccinated	2	1	-	–
	Not available	13	5	1	–
Clinical diagnosis of measles	No. of pts Time frame between evidence of measles and onset of ME (median, limits)	6 3 months (2–4)	4 2.4 months (1.2–4.2)	-	1 4 months
Time since presumed measles contact to SMME onset (mean, limits)		3.5 (1.4–5.8) months *N* = 12	5.4 (3.5–10) *N* = 6	N/A	4.2 months
Positive measles antibody at the moment of ME	IgM IgG	8 of 22 tested 12 of 14 tested	7 5	0 1	0 0
Measles qRNA detection from CSF		NA	5 of 6 tested	1	1
Median survival time since onset of ME (days) and range		18.5 (3–276)	25 (2–308)	Alive	9

Luminita Ene: LE

All except one child were born in the late 80s and are part of the Romanian HIV pediatric cohort and had epidemiological data suggesting parenterally non-IDU HIV transmission. The majority of patients were followed for HIV infection at VBH since their HIV diagnosis. Half of the patients had previously AIDS-defining diseases and the majority were severely immune suppressed. Six children had a CD4 count above 200, but only 4 of them had a CD4 percentage over 15. Only 20 of the 36 patients received combination antiretroviral therapy (cART); of them, 7, diagnosed during the first measles epidemic, received monotherapy or dual antiretroviral therapy. Further still, only 2 patients had undetectable plasma HIV RNA at the moment of neurological complication, but both with severe immune suppression. HIV RNA was measured in the CSF of 12 patients and was undetectable in 9 of them. The mean plasma HIV RNA values were 4.28 ± 1.23 (95%CI for a mean of 2.72–5.08), while mean CSF HIV RNA values were 2.50 ± 0.48 (95%CI for a mean of 2.23–3.27). CSF HIV RNA values were significantly lower (*p* < 0.0, *t* = 5.09) compared to plasma values.

Out of 17 patients with available medical records regarding vaccination, 14 children received at least one dose of vaccine against measles. Four children had evidence of measles infection during previous measles outbreaks (1992, 1995).

**FIGURE 1:**
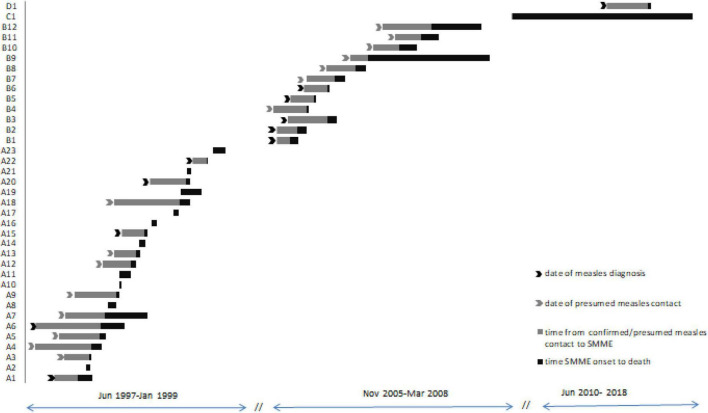
Timeline between measles episode or presumed contact with measles and SMME. Subjects given “A” numbers were diagnosed with SMME during the measles outbreak of 1997–1999, “B” numbers during the measles outbreak of 2005–2008, subject C1 was diagnosed in June 2010 and is a long-term survivor, and subject D1 was diagnosed in Jun 2017. Subjects A2, A8, and A18 had resilience at VBH during the measles epidemic.

We were able to establish the relationship between measles and SMME in 11 patients, and in 15 children presumed measles contact was identified within the median 4 months (range 1.4–10 months) before the onset of neurological complication (Figure 1). All children without a diagnosis of measles before SMME reported at least 1 episode of respiratory infection within 10 months before ME, but no skin or mucosal measles signs.

### Clinical findings

All patients shared a similar clinical presentation pattern. In most patients, clinical onset was sudden, in the absence of fever, with unilateral recurrent twitches (myoclonic jerks) localized on a body part. Myoclonic jerks extended rapidly (2–8 weeks) either involving other muscle groups from the same part of the body or from the opposite side. Myoclonic jerks had two patterns: one with continuous rhythmic muscular contractions enhanced by voluntary movements and slowly decreasing but still persistent during sleep compatible with the classical form of epilepsia partialis continua- EPC (ILAE Heinz Gregor Wieser) encountered in 17 patients, and a second episodic pattern with periods of continuous (varying from minutes to hours or days) myoclonus alternating with the absence of contractions, observed in 19 patients. The unusual location of myoclonic jerks was at the tongue in 9 patients or the presence of axial myoclonus in 4 patients. Episodes of generalized seizures and complex partial seizures overlap myoclonus in 15 patients. Motor impairment was absent or mild at the onset but weakness and plegia were noticed on initial myoclonic limbs afterward. Slurred speech and motor aphasia were diagnosed in 16 patients. Eight children had a somatosensory impairment, especially pain as the main complaint. Mental status was initially unaffected in 26 patients. Disease progressed over days or weeks to coma and death. Additional findings included visual impairment in 20 patients (12 with blindness, 8 with limitation of visual field), and visual and auditory hallucinations (4 patients).

### Laboratory data

Cerebrospinal fluid analysis was normal for most patients with regard to pleocytosis, albumin glucose, chloride level, and antibodies against CMV, Toxoplasma, EBV, rubella, HSV and venereal disease research laboratory were negative. PCR for JCV and tuberculosis DNA was negative However 13 children had mildly elevated albumin levels (0.66–1.65 g%_0_). Cultures of CSF for common bacteria, mycobacteria, and fungi were negative in all patients.

Measles virus antibody testing from both serum and CSF was performed inconstantly during the first epidemic, but systematically thereafter with inconclusive results. A couple of patients with clinical measles diagnosis and with MV isolated from pharyngeal swabs failed to produce measles antibodies.

Unfortunately, initial attempts to isolate MV from CSF failed, although for 3 patients from 2006 to 2008 and for one patient from 2010 to 2011 outbreak MV was isolated from pharyngeal secretions. In 2017 PCR from CSF and a nasopharyngeal swab was positive, with identical sequences on the measles virus nucleoprotein gene (N-450) indicating the B3 genotype prevalent in Romania (Lazar et al., 2019). The PCR performed on stored CSF samples 6 from 2006 to 2008 and 1 from the 2010 outbreak were positive for MV in all except one sample.

### Neuroimaging SMME

During the 1997–1998 outbreak CT scan was performed in 18 children: did not detect any abnormalities in 3 of them and identified hypointense cortical and subcortical areas without associated mass effect in 11 patients. Areas of hypodensity were either well defined–focal associated with continuous myoclonus pattern or small, patchy multilocular diffuse areas–associated with an episodic pattern of clinical myoclonus. Cortical atrophy in various degrees was described in 7 children, associated with hypointense lesions or as the only CT abnormality, in 4 children.

**FIGURE 2:**
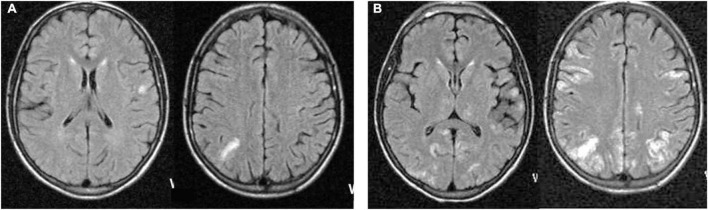
Magnetic resonance Fluid-attenuated inversion recovery (FLAIR) sequences from a representative patient at neurological presentation 3 months after a clinical measles episode demonstrating focal cortical gray matter cerebritis **(A)**, non-contrast-enhancing on T1 sequences (not shown), spreading 20 days later **(B)** to become multifocal and confluent, involving both hemispheres.

Seventeen patients underwent at least one MR imaging session at some point during the evolution of their illness. The most common (9/11 patients) early (within 3 weeks of initial presentation, typically with focal myoclonus) MR feature was uni- or oligo-focal cortical gray matter hyperintensity on T2 and fluid-attenuated inversion recovery (FLAIR) sequences. Among cases imaged early, radiographic lesion locations were consistent with the focality of their initial myoclonus in most. A minority of patients demonstrated additional lesions in the white matter of the cerebral hemispheres, typically adjacent to or confluent with gray matter lesions, or in the cerebellum. One case showed an initial subdural fluid collection over the right parietal occipital convexity, followed later by focal T2 hyperintensity lesions involving the cortical gray matter underlying the initial subdural lesion. In one patient, MR interpretation was confounded by previous cerebral toxoplasmosis that had responded to anti-toxoplasma therapy. No other patient demonstrated gadolinium-enhancing lesions or mass effects. Scans performed later in the course of illness, when multifocal myoclonus, epilepsia partialis continua, or encephalopathy occurred, usually demonstrated a bilateral hemispheric lesion distribution pattern (Figure 2). However, in three patients who survived SMME lesions seen on initial scans subsequently regressed. Overall, the most common imaging pattern was multifocal, spreading cerebritis.

In the five patients who underwent electroencephalography, background activity was predominantly slow, and epileptogenic discharges were seen in all. These discharges were typically bilateral and/or multifocal but in one case unifocal. The frequency of epileptogenic discharges appeared to correlate with the severity of clinical status. One patient had one of the highest CD4 counts in the cohort, and with treatment initiation, both clinical and electrographic states were observed to improve.

**FIGURE 3:**
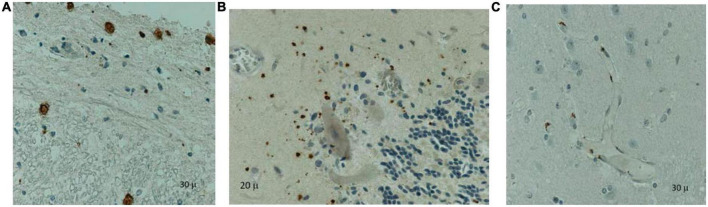
Immunohistochemistry on paraffin-embedded tissues using a measles monoclonal antibody (Chemicon, Sigma-Aldrich, Clones CV1, CV4) identified positive cells (brown staining) in the spinal cord between the gray and white matter and the meninges **(A)**, cerebellum **(B)** and cerebrum **(C)**: infiltrating macrophages and microglia in the deep gray matter. Counterstaining with hematoxylin.

*Histopathological evaluation* was available for 9 patients on postmortem brain tissue. Routine hematoxylin and eosin staining, and immunohistochemistry (IHC) examination were performed. All the slides stained for measles were positive. All the slides with cerebellum were positive by ICC for measles (Figure 3). The staining pattern was very similar in each case and varied only in the degree of abundance and intensity. The cerebral (gray and white matter) sections were positive, but the staining was significantly less abundant or intense. The IHC pattern suggests an ascending viral neural infection: the spinal cord is affected first and most, followed by the cerebellum and ultimately the cerebrum. Based on the cellular distribution, it seems that macrophages/microglia are a primary target. Still, the infection is not limited to this cell population; astrocytes and neurons may be infected too.

### Treatment and outcome

Antiepileptic drugs (Carbamazepine, Lamotriginum, Acidum valproicum, Phenytoinum, benzodiazepines) were unsuccessful in controlling myoclonic jerks. Antiviral treatment attempts were performed with Acyclovir, Foscavir, Ribavirin, or Interferon and also intravenous Immunoglobulin over time with no clinical improvement. Starting in 2006 all patients receive combination antiretroviral therapy (cART) soon after SMME diagnosis.

Despite all therapeutic approaches eventually, all except one patient died, with a median survival time from myoclonic jerk onset of 19 days. The mean survival period was longer (*p* = 0.007, *t* = 2.91) in the 5 patients with CD4 count above 200 cells/ml compared to those with severe immunosuppression: 38 days (95% CI 4.4–71.5) vs. 17 days (95%CI 13.3–22.1). Three patients had longer survival after the SMME episode. Two of them, diagnosed in 1997 and 2008, had an initial CD4 count above 200 cells/ml, and both had increased CD4 counts with cART. The patient from the first epidemic died after 125 days of sepsis with *Salmonella*. The other patient survived 308 days with the persistence of myoclonus, but with reduced intensity, she had a fair motor recovery being able to function autonomously. She stopped cART again, developed status epilepticus, and died rapidly in another center. The patient diagnosed in 2010 with SMME received a cART regimen including Abacavir, Lamivudine, Raltegravir, and Maraviroc, and is the only long-term survivor. He had excellent CD4 count recovery from 92 to 250 cells in 4 months period and to >500 cells/ml in one year period. He had residual mild right limbs motor deficit and impaired vision, without major interference with everyday living activity.

## Discussion

We describe here a particular form of SME that occurred in a group of parenterally infected children from Romania during consecutive measles epidemics and associated myoclonus as a pathognomonic clinical pattern. We emphasized that MV is responsible for this condition based on (1) the epidemiological context, (2) the temporal relationship between measles contact and the onset of myoclonus, (3) severe immune suppression related to HIV as an underlying condition, and finally (4) confirmatory diagnosis by positive immunocytochemistry for MV on brain tissues and/or presence of MV RNA in the CSF. As this entity was associated with severe immune suppression our team proposed it as an AIDS-defining disease (Duiculescu et al., 2007).

Subacute myoclonic encephalitis or measles inclusion body encephalitis has been previously described as a complication of measles in immune-compromised patients due to HIV (Mustafa et al., 1993, Vigliano et al., 1995, Poon et al., 1998) or other medical conditions (Wolinsky et al., 1977, Gazzola et al., 1999, Caraballo et al., 2003), (Freeman et al., 1967, Esiri et al., 1982). Measles virus was isolated from the brain of patients with SME as inclusions, and measles antibodies were absent or inconstant in CSF (Freeman et al., 1967, Mustafa et al., 1993). A case series of 8 HIV-infected patients with SMME has been described in a tertiary hospital in South Africa in 2010, and similarly to our observation reflects the possibility of clusters of SMME during measles outbreaks in severely immune-suppressed HIV-infected individuals (Albertyn et al., 2011). Although the earlier case reports mentioned altered mental status and lethargy as presenting symptoms, our similarities to the other case series suggest that localized myoclonus or epilepsia partialis continua is a pathognomonic sign and mental status is initially preserved. Based on all these characteristics we proposed to name this clinical entity subacute myoclonic measles encephalitis.

Even though rare, myoclonus as a clinical sign of neuronal injury has been described in HIV-infected individuals, It was linked to HIV itself in acute infection (Denning, 1988), HIV-associated dementia (Canafoglia et al., 2003), to HIV immune reconstitution syndrome (Kanjanasut et al., 2010, Pereira et al., 2016) or HIV viral escape in CSF (Cabaraux et al., 2020). Neurological HIV-associated opportunistic infections such as cerebral toxoplasmosis (Mattos et al., 2002), progressive multifocal leukoencephalopathy (Bettoni et al., 1986), or brain tuberculosis (Guedes et al., 2018) could also present with myoclonus. There was however so far no pattern of myoclonus as we described for SMME in our HIV group.

The most common neurological complication of measles is subacute sclerosing panencephalitis (SSPE), which occurs after years from initial exposure to MV (mean 7–12 years). The pathogeny of SSPE is linked to the accumulation of a mutated virus in the brain tissue (Norrby and Kristensson, 1978), and this results also in high titers of measles antibodies in CSF. There are some remarkable differences encountered in our patients in comparison to those with SSPE. First, the short time frame from measles episode or contact to neurological debut. Secondly, from a clinical point of view, our patients had initially preserved mental status and motor involvement i.e., myoclonus was the first manifestation. The initial lesions in the brain of the patients with SSPE are in the cortical gray matter progressing to subcortical gray and white matter. The MRI lesions in our patients suggest also initial involvement of cortical gray matter and consecutive extension to the subcortical gray and white matter but compared to SSPE the extension of the lesions is faster and the lesions are not so wide. Neurological progression is faster in our group with a median time to death of 3 weeks compared to years in SSPE. We were not able to describe a pattern of measles antibody presence or dynamics, probably due to altered immune responses consecutive to severe immune suppression.

A fulminant pattern of SSPE has been previously described in two children and an adult without an identified cause of immune suppression (Kravljanac et al., 2011, Garg et al., 2017). All had epilepsia partialis continua (EPC) and died within 3 months from onset. Still, these children had initial cognitive decline and EPC was present afterward. Another fulminant course of SSPE has been described in a newborn who acquired measles intrauterine (Dasopoulou and Covanis, 2004), and the rapid course could have been driven by the absence of immune responses.

To answer the question of whether SMME could be a fulminant course of SSPE in severely immunosuppressed children, as previously suggested (Kravljanac et al., 2011), there are several aspects to consider. Our SMME patients shared similarities with fulminant cases of SPPE: HIV infection as a risk factor for altered immune responses (Manesh et al., 2017), similar ICC pattern of an ascending viral neuronal infection, and although less commonly, presence of high IgG titers of measles antibodies in CSF and rapid progression time to death (Kravljanac et al., 2011, Magurano et al., 2017). However, there are distinct aspects between SMME and SSPE: the shorter time interval between the measles episode or contact with measles and the onset of neurological signs, the lack of intellectual impairment at onset as described in SPPE (Dyken, 1985), the lack of typical SSPE patterns in EEG examination (even though this has only been performed for a few patients with SMME). SPPE patients have MV isolated from CSF with multiple mutations in the M and F proteins, affecting its fusogenic capacity and facilitating the transsynaptic spreading (Shirogane et al., 2023), while we found genetically similar MV in CSF and nasopharyngeal swabs from the SMME patient. Also, as SMME has occurred in severely immunosuppressed HIV individuals, fulminant SSPE has been reported in HIV-infected individuals with good CD4 counts (Sivadasan et al., 2012; Maurya et al., 2016, Vidhale et al., 2021). It has been hypothesized that fulminant SSPE may be another example of an inflammatory immune reconstitution syndrome as a paradoxical exacerbation of a latent infection with mutant MV (Gupta et al., 2017).

One of the key features associated with SMME is severe immune suppression, present in almost all patients described here. Our medical data suggest that most patients had received at least one dose of the measles vaccine, and some of them had been previously diagnosed with measles. Altered humoral responses (Moss et al., 1999) in the setting of immune suppression due to HIV, can explain the absence of response to vaccination or rather a loss of the initial response to the measles vaccine. Previous studies reported that the antibody response to vaccination in children with HIV infection varies between 25 and 37% after the first dose of vaccine to 50–66% after the second dose (Moss et al., 1999). In a personal communication, the proportion of HIV-infected children with IgG antibodies was 23.33% after the first dose of vaccine and 27.3% for children who received two doses of measles vaccine (Duiculescu et al., 2006). The absence of memory cells after measles vaccination has been reported even in HIV-infected children with good immunological reconstitution on cART (Aurpibul et al., 2006, Abzug et al., 2012). The absence or atypical measles manifestation prior to SME is linked also to impaired immune responses (Markowitz et al., 1988, De Carvalho et al., 2003), and makes the association between measles and neurological presentation even more difficult.

Measles virus was found in all brain tissues from the 9 patients with available brain tissues, still *in vivo* diagnosis is often difficult. The initial attempts to isolate MV from the CSF of the patients with SMME were not successful. We assumed that the virus from the brain of the patients with SMME was different from that of pharynges and therefore couldn’t be detected with the same primers or that CSF could have several inhibitor substances. However, in 2017 MV RNA was detected by PCR from CSF, and was positive on CSF from stored samples from previous epidemics. As some of the CSF samples were tested with several assays, we assume the latest one was more sensitive. Moreover, for the last patient diagnosed in 2017, MV isolated from the nasopharynx was similar to that isolated from the CSF. There are conflicting data from the literature regarding the genetic profile of MV responsible for the measles inclusion body encephalitis. In one of the first communication on this entity in 1981, Roos et al. (1981) found mutated M protein measles in a child with MIBE implying similitudes with PESS and concludes that the pathogenesis of both neurological entities is similar. In another study, the matrix protein was present in the brain, cerebrospinal fluid, and serum and was demonstrated by Western blot analysis and *in situ* hybridization in a patient with MIBE (Kim et al., 1992). In a case report of a patient without any remarkable immune suppression diagnosed with MIBE, MV was isolated from brain tissue, spinal cord, and eye, but not from CSF (Croxson et al., 2002). In another study on 4 patients with MIBE and HIV diagnosed during the last measles epidemics from South Africa (2009–2010), the authors were able to isolate MV from both CSF and brain tissue and to demonstrate by phylogenetic analysis a similar profile with the circulating virus, with only one noticeable exception namely the mutation L454 W in the fusion protein that was found at 2 of the 4 patients (Hardie et al., 2013). Following this report, it was hypothesized that a potentially neuropathogenic variant might emerge outside the CNS, can infect new hosts via the respiratory route, and is more pathogenic (in animal models) (Mathieu et al., 2019). Another published case report of a Romanian young woman with AIDS diagnosed with measles inclusion body encephalitis in 2018 did not however find the mutations previously associated with neurovirulence (Rodriguez et al., 2020).

Immunocytochemistry data from patients with available brain tissue suggests an ascending pattern of the infection with MV, with the most abundant infection in the spinal cord rather than in the cerebellum, and finally in some areas of the cortical tissue. Based on the cellular distribution of MV, the target cells seem to be macrophages and microglia, but the infection is not limited exclusively to these cells, targeting also neurons and astrocytes. In a very interesting study on cerebral tissues from children with HIV infection that died from measles or measles-related complications, McQuaid et al. (1998) detected MV in one patient with measles encephalitis. MV was found in neurons, oligodendrocytes, microglia, and almost all cortical areas with similar distributions to the patients with SSPE. Therefore the authors conclude that HIV might induce an increased susceptibility to CNS infection of MV (McQuaid et al., 1998).

Neuroimaging exams localized gray matter cortical abnormalities with rapid extension. The location of the lesions described in other patients with MIBE does not appear to have a distinct pattern. Neuroimaging lesions from patients with MIBE were located at basal ganglia, temporal and occipital areas (Drysdale et al., 1976), frontal (Freeman et al., 1967), and occipital (Gazzola et al., 1999) areas. In patients surviving SMME episodes, the MRI lesions seem to regress over time, and this aspect has been reported in HIV (Albertyn et al., 2011) and non-HIV patients recovering immune suppressive conditions (Chong et al., 2007).

Does the immune reconstitution induced by controlling HIV replication with cART activate immune responses that would be able to control the latent infection with MV similar to other infections (i.e., progressive multifocal leukoencephalopathy)? This latest idea is supported by the observation that patients who survived SMME had potent cART. Also, wide access to cART might has contribute to the reduced number of patients diagnosed during the latest measles outbreaks.

The particularities of SMME in our group of patients have to be examined from the perspective of the interactions of both MV and HIV. MV induces an impairment of cellular immunity by the cumulation of several mechanisms as interference with cytokine synthesis. In MV and HIV co-infected patients, T cell reduction has two mechanisms, becoming thus a supplementary factor for opportunistic infections, but also for the dissemination of the MV to the CNS through the nervous tracts. An inhibitory effect of MV on HIV replication during acute measles (Moss et al., 2009) has been described. Studies on lymphoid human tissue infected *ex vivo* with HIV-1 and MV demonstrated that the most inhibited was HIV R5 tropic virus. MV inhibits the replication of R5 HIV-1 in coinfected tissues by up-regulation of the CC chemokine RANTES, a well-known inhibitor of R5 HIV-1 infection, and this up-regulation is augmented in tissues coinfected with R5 HIV-1 (Grivel et al., 2005). Until now the inhibitory effect of MV on HIV has been described in plasma during acute measles infection. We have found undetectable HIV loads in CSF in 8 of 10 patients tested, while the other two had also HIV RNA CSF loads below <400 c/ml. Out of these patients, only 2 had undetectable HIV RNA in plasma. The other patients were on failing cART or without antiretroviral treatment. The low HIV RNA loads in CSF might be interpreted as the result of the suppression of HIV replication in CSF by MV during SMME. This aspect has been reported only by our group until now (Ene et al., 2007).

Another interesting aspect is the number of patients diagnosed with SMME over time. If during the first described measles outbreak some cases might have been overlooked, during the next outbreaks, the number of cases decreased, even if physicians became aware of this condition. One explanation could be that the incidence of measles in Romania decreased after 2000 and during the last outbreaks, the affected population consisted mainly of small children with no or incomplete vaccine coverage (Dascalu, 2019). Also, access to more efficient antiretrovirals and improved adherence might have decreased the number of patients with severe immune suppression at risk to develop SMME during the latest measles outbreaks.

Our study had several limitations. Due to the low frequency, initially, it was difficult to link the neurological complication to measles, and therefore we could have missed some patients during the first measles outbreak. Second, we selected the patients based on the myoclonus as a pathognomonic pattern, so we could also miss some cases that could present without myoclonus, especially since there was impossible to have a molecular diagnosis.

In conclusion, subacute myoclonic measles encephalitis can be suspected in an HIV-infected patient with severe immune suppression presenting with myoclonus and initially preserved mental status during a measles epidemic. Prevention and treatment methods are warranted in order to suppress MV in the brain, in association with cART in order to restore immunity, especially in limited-resource countries.

## Data availability statement

The raw data supporting the conclusions of this article will be made available by the authors, without undue reservation.

## Ethics statement

The studies involving human participants were reviewed and approved by the Ethics Committee of “Dr. Victor Babes” Hospital for Infectious and Tropical Diseases Bucharest Romania. Written informed consent from the participants’ legal guardian/next of kin was not required to participate in this study in accordance with the national legislation and the institutional requirements. Written informed consent was obtained from the individual(s) for the publication of any potentially identifiable images or data included in this article.

## Author contributions

DD designed the study, conducted a follow-up of patients and investigations, and presented early data. LE designed the study, collected and analyzed the data, and drafted the manuscript. RR and EU collected the data. ML, GT, and SR performed virological studies. HV and CA performed histological studies. RE contributed to neurological case description and neuroimaging analysis. LE, RR, ML, GT, EU, SR, HV, SL, IG, RE, and CA reviewed the manuscript and approved the final version of the manuscript. All authors contributed to the article and approved the submitted version except DD (deceased).
